# Generation of stable homozygous transformants of diploid yeasts such as *Xanthophyllomyces dendrorhous*

**DOI:** 10.1007/s00253-022-12054-2

**Published:** 2022-07-14

**Authors:** Gerhard Sandmann

**Affiliations:** Institute for Molecular Biosciences, Department of Bio Sciences, Goethe University Frankfurt, Frankfurt/M, Max von Laue Str. 9, 60438 Frankfurt, Germany

**Keywords:** Carotenoid biosynthesis, Ploidy, Genetic engineering,Heterozygosity,Mitotic recombination, Sexual cycle

## Abstract

**Abstract:**

The nonconventional yeast *Xanthophyllomyces dendrorhous* is an established platform for genetic pathway modification. A genetic tool box is available and can be used extensively to select from for different engineering strategies. Due to the diploid nature of *X. dendrorhous*, genetic transformation typically results in heterozygous lines. They are genetically unstable and lose their phenotypes caused by mitotic recombination. In addition, targeted integration for inactivation of genes of the carotenoid pathway resulted in an intermediary phenotype of incomplete pathway disruption. This issue is the main scope of this review. It is illustrated by using genetic modification of the carotenoid pathway of *X. dendrorhous* as a model system with a focus on the demonstration of how to solve these problems by generation of homozygous lines. They can be selected from heterozygous transformants after spontaneous mitotic recombination and selection or after induced meiotic recombination. Corresponding methods of how to proceed including the initiation of a sexual cycle are described. The selected segregated lines are stable in fermenter cultures without the need of selection pressure. This is an essential requirement for any industrial application.

**Key points:**

*• Genetic interventions of diploid yeasts result in heterozygous transformants that are unstable without selection pressure.*

*• This is due to mitotic recombination leading to the elimination of inserted DNA.*

*• Stable homozygous lines can be obtained and selected after either meiotic or mitotic recombination.*

## Introduction

*Xanthophyllomyces dendrorhous* is a heterobasidiomycetous budding yeast belonging to the order Cystofilobasidiales (Webster and Weber 2007). Different strains were isolated from exudates of beach or birch trees (Libkind et al. 2007). This nonconventional yeast was first described as *Rhodozyma montanae* and later renamed as *Phaffia rhodozyma* in its anamorph form (Golubev 1995). The perfect state, *X. dendrorhous*, is homothallic and grows diploid in its vegetative phase (Kucsera et al. 1998; Webster and Weber 2007). A sexual cycle can be initiated by cultivation with polyols as sole carbon source combined with nitrogen limitation. It involves cell fusion to a tetraploid zygote and formation of a holobasidium with diploid spores after meiosis (Golubev 1995).

*X. dendrorhous* is of special interest since it is the only fungus able to synthesise the carotenoid astaxanthin, which is of great commercial value (Sandmann 2014). Astaxanthin is a powerful antioxidant and a feed additive for pigmentation in salmon farming. This application promoted the development of *X. dendrorhous* as a platform for the production of lipophilic carotenoids by genetic pathway intervention. In addition to the engineering of strains for high-yield astaxanthin synthesis and other valuable carotenoids (Sandmann et al. 2021), *X. dendrorhous* was genetically modified for the synthesis of α-cuprenene (Melillo et al. 2013) and long-chain polyunsaturated fatty acids (Sandmann et al. 2021). In recent years, proteomic, transcriptomic, and metabolomic techniques, reviewed by Barredo et al. (2017), including metabolic profiling (Alcalde and Fraser 2018) have been adapted to *X. dendrorhous* which is useful to analyse metabolic variations affecting other unrelated pathways in these mutants and in the genetically modified strains.

Under selection pressure, transformants of *X. dendrorhous* are stable retaining their genetic modification and their phenotype. However, in fermenter cultures without the selection agents, cells revert fast to the wild-type phenotype and overgrow the transgenic cells. This effect originates from the heterozygosity of the transformants of this diploid yeast strain and is caused by a high mitotic recombination rate (Niklitschek et al. 2008; Pollmann et al. 2017a). Mitotic recombination also affects the stability of classical mutants due to their heterozygosity (Medwid 1998; Sandmann et al. 2021). This process is a disadvantage for the utilisation of the generated and selected astaxanthin hyper-producing mutants (Torres‑Haro et al. 2021). In addition to instability, gene inactivation failed to result in the desired complete pathway disruption.

*X. dendrorhous* is the only diploid yeast for which the problems of instability and insufficient pathway disruption arising in the course of genetic engineering have been pointed out. Recent reviews on carotenoid synthesis of genetically engineered microorganism including *X. dendrorhous* have ignored this aspect (Li et al. 2020; Zhang et al. 2020). Therefore, this review is focussed on the problems encountered on genetic engineering of the carotenoid pathway of diploid *X. dendrorhous*. It also includes and illustrates different procedures to generate stable transformants in the absence of selection agents by converting the heterozygous into homozygous lines, which should also be applicable for other diploid yeasts. This is a precondition for high-density fermenter cultures necessary for industrial production of interesting compounds from engineered *X. dendrorhous* strains.

## Strategies for genetic pathway engineering with *X. dendrorhous*

*X. dendrorhous* was primarily used to engineer the carotenoid pathway in order to obtain different commercially interesting carotenoids. This was achieved with different strategies: the increase of existing carotenoid synthesis by pathway enhancement, accumulation of intermediated of the pathway, or the generation of novel structures by pathway extension (Sandmann et al. 2021).

For genetic modification of *X. dendrorhous*, all necessary genetic techniques and tools are available. A protocol for efficient transformation by electroporation has been published (Visser et al. 2005). Genes have been integrated into the rDNA of the genome with suitable plasmids. The first four different plasmids were constructed with different selection markers (Gassel et al. 2014). Each of them is able to carry and insert two individual genes under strong constitutive promoters. Theoretically, up to 61 copies of this type of plasmid can be integrated (Wery et al. 1997). Two other integrative plasmids for the transformation of *X. dendrorhous* include one or three sites for insertion of genes under different promoters (Hara et al. 2014). The availability of the genome sequences of two *X. dendrorhous* strains (Sharma et al. 2015; Bellora et al. 2016) helps to identify target genes for knockout involved in pathways to be modified.

The astaxanthin concentrations of *X. dendrorhous* wild-type strains of around 200 µg/g dry weight (Sandmann 2014) are too low for commercial exploitation. Therefore, molecular genetic modification aimed at the increase of astaxanthin synthesis by overexpression of genes of limiting pathway enzymes. Among them were the gateway enzymes of the general terpenoid pathway and of the specific carotenoid pathway (Gassel et al. 2014). Alternatively, three genes of the mevalonate pathway, which provides the precursors for carotenoid synthesis to improve astaxanthin production, were overexpressed (Hara et al. 2014).

The *X. dendrorhous* transformant with enhanced carotenoid metabolism mentioned above was utilised continuously for the accumulation of carotenoid pathway intermediates by gene inactivation (Niklitschek et al. 2008). By homologous recombination with a cassette with sequences of the target gene and a selection marker in this gene, the phytoene desaturase gene was knocked-out (Pollmann et al. 2017a). Blocking the pathway at this stage, the carotene phytoene becomes the end-product of the truncated pathway. This approach can be advanced by knocking-in a gene of interest into the target gene to be knocked-out (Breitenbach et al. 2019). It was used for the extension of the carotenoid pathway of *X. dendrorhous* from accumulating β-carotene to the formation of zeaxanthin, which was followed further on by transformation with a combination of several genes for the synthesis of novel multi-oxygenated carotenoids (Pollmann et al. 2017b). It is also possible to eliminate selectable markers from the *X. dendrorhous* genome by the Cre-loxP system as shown recently (Zhang et al. 2019).

## Ploidy of *X. dendrorhous* and its effect on transgene stability

**Fig. 1:**
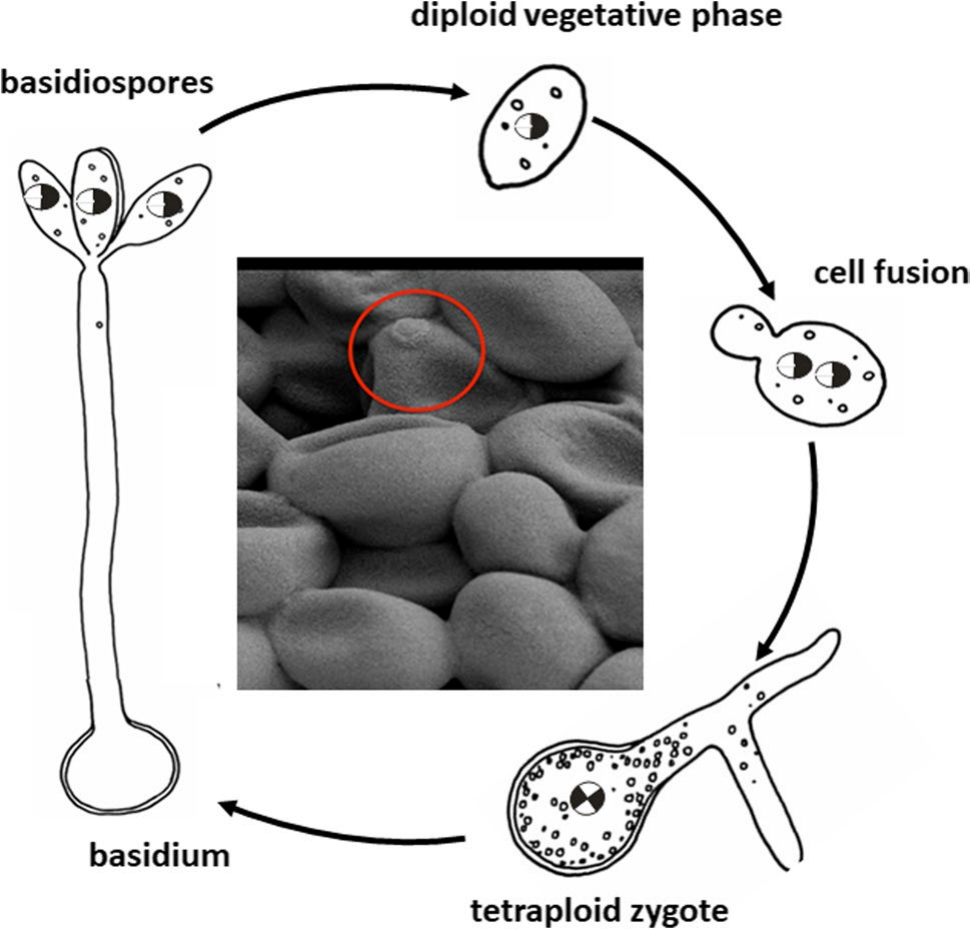
Sexual cycle of the diploid and homothallic yeast *Xanthophyllomyces dendrorhous* (adapted from Webster and Weber 2007) starting from fusion of diploid vegetative cells to form a tetraploid zygote and meiotic transformation to diploid basidiospores, which germinate, into vegetative growing cells. The centre shows a scanning electron-microscopic picture of a budding cell (red circle)

For the stability of a genetically modified transformant and of the engineered phenotype, the ploidy of the recipient organisms is decisive. The ploidy of *X. dendrorhous* was initially regarded as haploid (Wery et al. 1997) but this was ruled out by Medwid (1998). The haploid nature of *X. dendrorhous* was further challenged by a spore segregation experiments by Kucsera et al. (1998) which pointed at diploidy. This result led to the establishment of a life cycle for homothallic *X. dendrorhous* with a vegetative diploid phase, cell fusion to a tetraploid zygote, and formation of a holobasidium where meiosis generates diploid basidiospores (Fig. 1). Finally, these spores germinate to vegetative cells, which multiply by budding.

The most direct results unequivocally demonstrating a diploid nature were obtained from knock-out experiments of genes of the carotenoid pathway of strain ATCC 24230 initially resulting in transformants with an unchanged phenotype (Niklitschek et al. 2008). This finding was further substantiated with inactivation of two carotenogenic genes (Pollmann et al. 2017a; Breitenbach et al. 2019) in *X. dendrorhous* strain CBS6938 resulting in an intermediate phenotype. In both cases, the presence of different allele sizes has been shown demonstrating that both transformed strains were heterozygous. This was also the case after integration of a bacterial gene to extend the carotenoid pathway (Pollmann et al. 2017c). A consequence of the diploid character of *X. dendrorhous* is the formation of heterozygous transformants (Hermosilla et al. 2003). This situation promotes the loss of integrated trans genes by non-reciprocal DNA transfer between homologous chromosomes resulting in a decreasing carotenoid production.

## Mitotic recombination: disadvantage and advantage for stability of transgenic lines

**Fig. 2:**
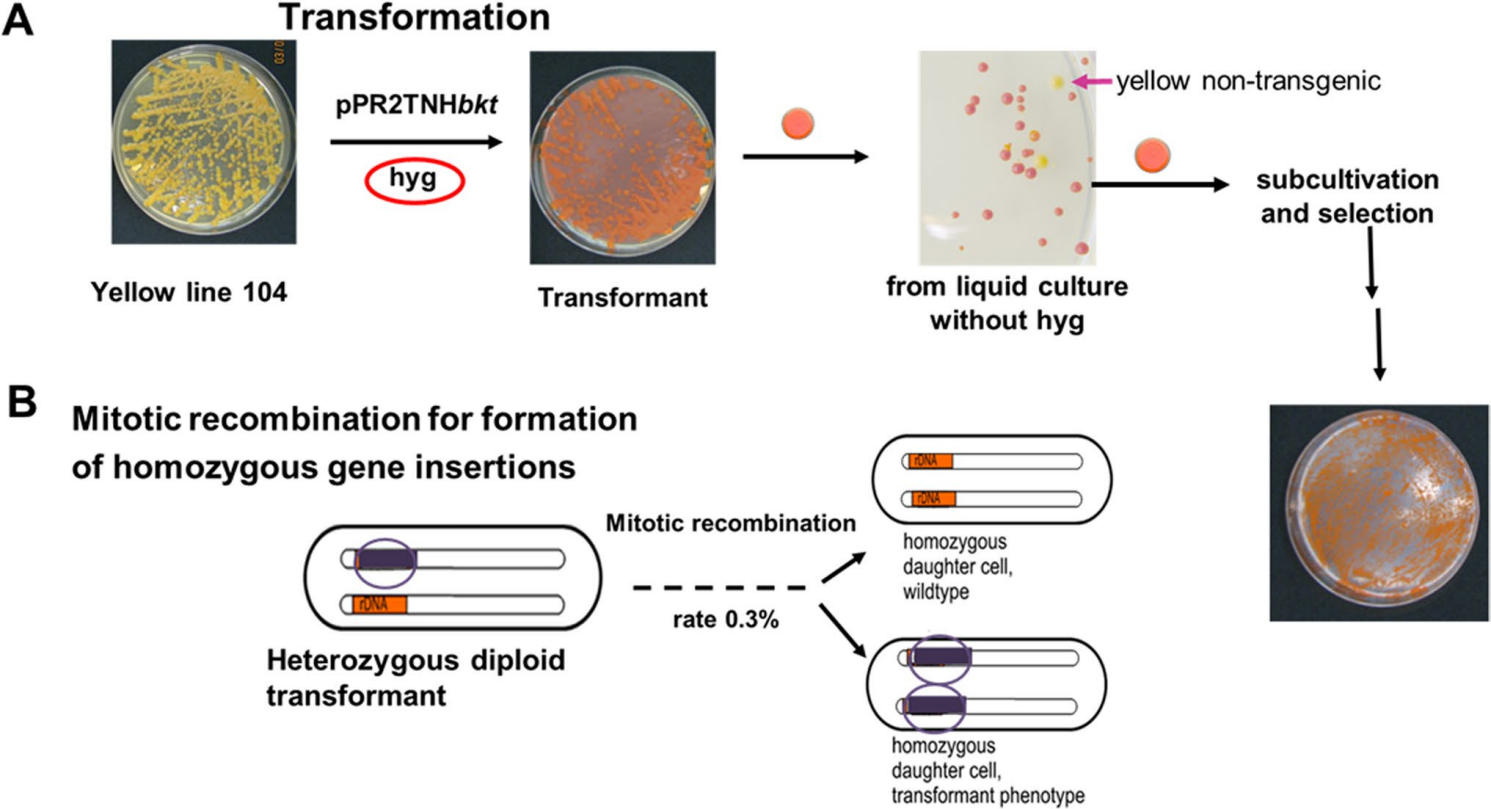
Mitotic recombination affecting transformants of *Xanthophyllomces dendrorhous.* **A** Transformation of a yellow mutant and selection of red transformants with hygromycin; growth of a red cells in the absence of hygromycin resulting in a mixed culture with red and yellow colonies; repeated sub-cultivation and selection of the red phenotype yields homogenous stable cultures, in analogy to Breitenbach et al. (2019). **B** Illustration of mitotic recombination of the heterozygous diploid *X. dendrorhous* transformant and segregation into homozygous wild type with the transgenic phenotype

Mitotic recombination is a mechanism for DNA reconstitution, which is well documented in yeasts (Prado et al. 2003). During mitosis in vegetative diploid cells, chromosomal exchange between homologous DNA sequences located at allelic positions in homologous chromosomes occurs resulting in loss of heterozygosity (Jinks-Robertson and Petes 2021). This process eliminates mutations and integrated exogenous DNA from the genome, which in the case of the *X. dendrorhous* transformants are the cassettes with the integrated carotenogenic and antibiotic resistance genes. This event is demonstrated in Fig. 2: A yellow β-carotene accumulating *X. dendrorhous* strain was transformed with a cassette containing an astaxanthin synthase and a hydromycin-resistance gene. In the presence of the selection agent, red astaxanthin-accumulating transformants survive exclusively. However, omission of hygromycin from a culture originating from a red transformant leads to the enrichment of yellow non-transgenic cells, especially in fermenter cultures where they more and more overgrow the transgenic cells. Reversion of the transgenic status is caused by mitotic homologous recombination due to the two different templates of the heterozygous line, one of genetically modified genomic region and one of the corresponding wild-type allele (Dutta et al. 2021). The rate for mitotic recombination of the inactivated phytoene desaturase gene *crtI* in *X. dendrorhous* of 0.3% (Pollmann et al. 2017a) is comparably high (Thornton and Johnston 1971). In addition, homozygous transgenic cells are also generated. In general, mitotic recombination is not only an obstacle causing a vanishing phenotype but can also be used for the selection of stable transgenic lines. In the example of Fig. 2, this was achieved after plating the transformant on a non-selective medium and picking a red colony, which may be either heterozygous or homozygous. Repeated plating of red colonies will finally result in a plate with red colonies exclusively, which resemble homozygous lines. This procedure was successful in the generation of stable transgenic lines (Niklitschek et al. 2008) applicable for the production of carotenoids in fermenter cultures (Pollmann et al. 2017a). An alternative way to select homozygous lines of transgenic *X. dendrorhous* succeeding mitotic recombination is by growth with increasing concentration of the selection agent (Niklitschek et al. 2012). By application of this procedure following inactivation of the C-22 sterol desaturase gene, only homozygous lines with both disrupted alleles finally survived (Yamamoto et al. 2016).

## Sexual cycle for the generation of homozygous transformants

**Fig. 3:**
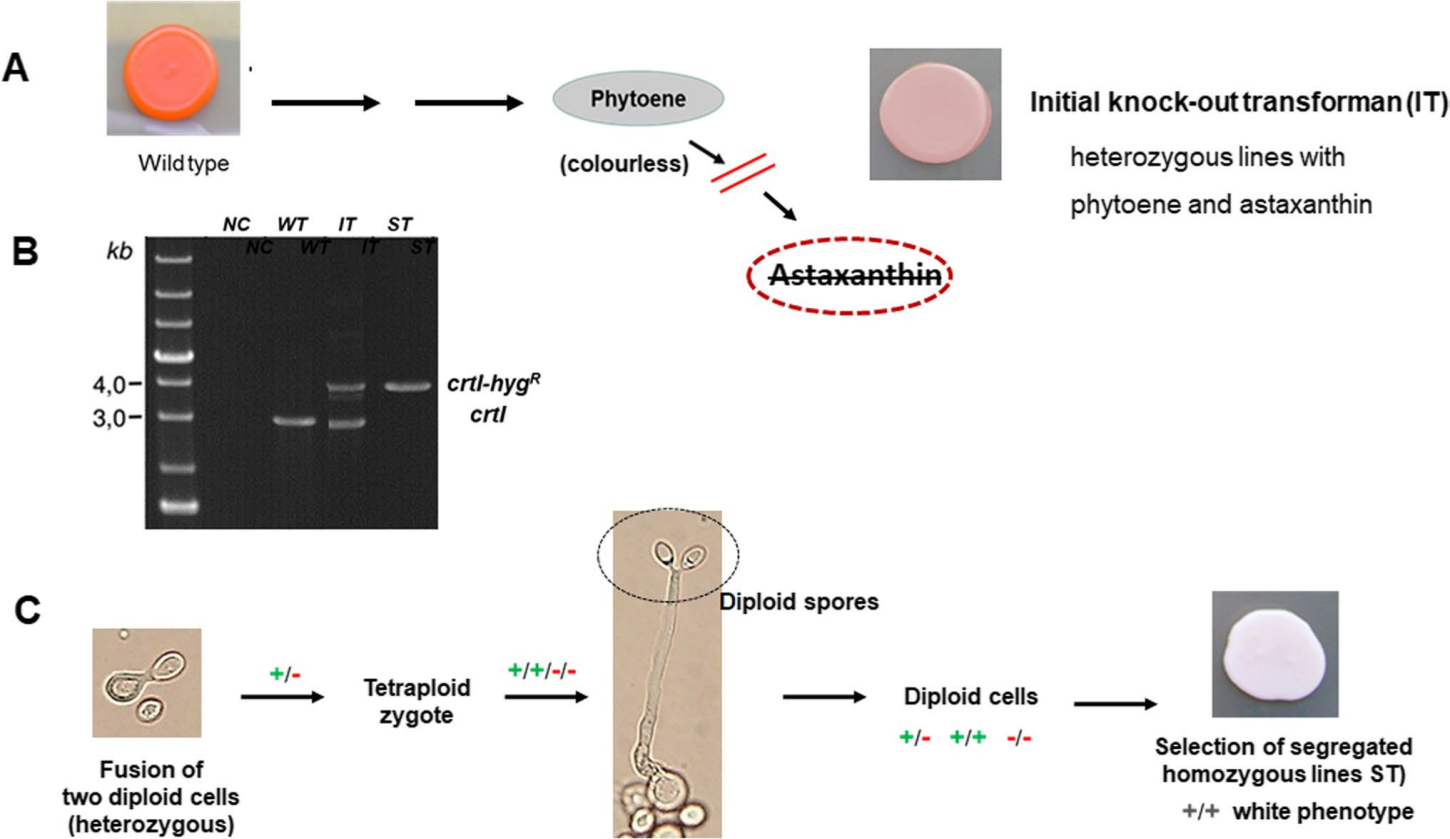
Formation of an intermediate phenotype due to heterozygosity after gene inactivation of *Xanthophyllomces dendrorhous* and generation of homozygous lines through a sexual cycle corresponding to Pollmann et al. (2017c). **A** Inactivation of the gene of the phytoene-metabolising enzyme, *crtI*, resulting in yellowish transformants (IT). **B** Demonstration of the presence of the wild type together with the inactivated gene (lane IT) in the transformant. **C** Different stages of the sexual cycle of the diploid heterozygous transformant and selection of segregated, white lines (ST) which are homozygous for the inactivated gene (part B, lane ST)

A specific problem of gene inactivation emerges in diploid yeasts: Both copies of the gene have to be inactivated to avoid heterozygosity resulting otherwise in incomplete pathway disruption. An example is illustrated in Fig. 3. After transformation targeting the phytoene desaturase gene *crtI* involved in phytoene conversion, the initial knock-out transformant (IT) contains not only colourless phytoene in contrast to the wild type but also astaxanthin, the end-product of the pathway, as indicated by its yellowish pigmentation (Fig. 3A). The separated amplified DNA of this culture exhibits two *crtI*-related bands, the intact *crtI* gene (also present in the wild type) and *crtI* with the inserted resistance gene (Fig. 3B). For complete blockage of phytoene conversion and its exclusive accumulation, this demonstrated heterozygous state of *crtI* has to be changed to homozygosity. As in Fig. 2B, a wild-type and a knocked-out allele has also been obtained in each case when several other carotenogenic genes were inactivated (Niklitschek et al. 2008).

A promising way for getting homozygous transgenic transformants is self-mating. This can be initiated with the homothallic *X. dendrorhous* by application of ribitol as the exclusive carbon source and growth on plates at 10 °C (Golubev 1995). The details of how to induce the sexual cycle are described in Pollmann et al. (2017c). Two heterozygous diploid cells fuse to a tetraploid zygote. After meiosis, a holobasidium with diploid basidiospores is formed (Fig. 3C). Segregation produces diploid basidiospores, which are either homozygous for *crtI*, homozygous for inactivated *crtI* with the inserted hyg^R^ cassette, or heterozygous. Germinating basidiospores were collected and after growth white colonies selected (Fig. 3C). In the segregated lines of the white phenotype (ST), the *crtI* band is absent (Fig. 3C) demonstrating their homozygous state. In fermenter cultures, these homozygous phytoene-accumulating transformants were stable for more than 20 generations under production conditions in the absence of selection pressure (Sandmann et al. 2021).

Another strategy for complete gene inactivation in a diploid yeast is by consecutive double transformation to target each allele with insertion of different selectable markers. This approach was used to inactivate the asy gene (Breitenbach et al. 2019) in *X. dendrorhous* and to modify its sterol pathway (Loto et al. 2012). The latter approach resulted in transformants, which were homozygous for the inactivated sterol desaturase gene but heterozygous for both selection markers. There is nothing known about the long-term stability of this type of transformants.

## Conclusion

The carotenoid metabolism of diploid *X. dendrorhous* was genetically engineered by gene integration to overcome limiting steps in the pathway and by gene inactivation to accumulate pathway intermediates. Integration of exogenous DNA into a diploid yeast produces heterozygous transformants. As exemplified for *X. dendrorhous*, this heterozygosity causes instability of the trait by loss of the inserted DNA due to mitotic recombination. Additionally, in the case of gene knockout of diploids, the non-targeted allele prevents complete pathway disruption. It has been outlined in this review how both these problems can be solved by making transformants homozygous: Stable phenotypes can be obtained either by selection of homozygous transformants emerging from spontaneous mitotic recombination or by meiotic recombination after initiation of a sexual cycle. The generated homozygous lines with an inactivated pathway gene also offer the advantage of a complete termination of a reaction sequence targeted for the accumulation of a metabolic intermediate.

Genetic pathway engineering of so far unnoticed nonconventional yeasts including diploid species offers promising opportunities for new industrial production processes. The described methods for obtaining homozygous transformants are applicable in combination with any genetic intervention of diploid yeasts. They may be specifically useful for diploid strains of *Rhodosporidium toruloides* (also known as *Rhodotorula toruloides*) (Liu et al. 2017) and *Candida utilis* (Liang and Bennett 2020) which similar to *X. dendrorhous* are already of interest for genetic engineering of the carotenoid biosynthesis pathway (Shimada et al. 1998; Wen et al. 2020) or for diploid dairy isolates of *Kluyveromyces marxianus* (Ortiz-Merino et al. 2018). A future application of obtaining homozygous transgenic may be the CRISPR/Cas9 system for gene-specific genome interventions (Hsu et al. 2014). This technique has been used for integration of carotenogenic genes into *Yarrowia lipolytica* (Schwartz et al. 2017). A first attempt applying this technique to *X. dendrorhous* has been successful (Hong et al. 2021). Future developments may demonstrate its potential in pathway modifications of *X. dendrorhous* and other diploid yeasts.
